# SOAPdenovo2: an empirically improved memory-efficient short-read *de novo* assembler

**DOI:** 10.1186/2047-217X-1-18

**Published:** 2012-12-27

**Authors:** Ruibang Luo, Binghang Liu, Yinlong Xie, Zhenyu Li, Weihua Huang, Jianying Yuan, Guangzhu He, Yanxiang Chen, Qi Pan, Yunjie Liu, Jingbo Tang, Gengxiong Wu, Hao Zhang, Yujian Shi, Yong Liu, Chang Yu, Bo Wang, Yao Lu, Changlei Han, David W Cheung, Siu-Ming Yiu, Shaoliang Peng, Zhu Xiaoqian, Guangming Liu, Xiangke Liao, Yingrui Li, Huanming Yang, Jian Wang, Tak-Wah Lam, Jun Wang

**Affiliations:** 1 BGI HK Research Institute, 16 Dai Fu Street, Tai Po Industrial Estate, Hong Kong; 2 HKU-BGI Bioinformatics Algorithms and Core Technology Research Laboratory & Department of Computer Science, University of Hong Kong, Pokfulam, Hong Kong; 3 School of Bioscience and Bioengineering, South China University of Technology, Guangzhou, 510006, China; 4 School of Computer Science, National University of Defense Technology, No.47, Yanwachi street, Kaifu District, Changsha, Hunan, 410073, China

**Keywords:** Genome, Assembly, Contig, Scaffold, Error correction, Gap-filling

## Abstract

**Background:**

There is a rapidly increasing amount of *de novo* genome assembly using next-generation sequencing (NGS) short reads; however, several big challenges remain to be overcome in order for this to be efficient and accurate. SOAPdenovo has been successfully applied to assemble many published genomes, but it still needs improvement in continuity, accuracy and coverage, especially in repeat regions.

**Findings:**

To overcome these challenges, we have developed its successor, SOAPdenovo2, which has the advantage of a new algorithm design that reduces memory consumption in graph construction, resolves more repeat regions in contig assembly, increases coverage and length in scaffold construction, improves gap closing, and optimizes for large genome.

**Conclusions:**

Benchmark using the Assemblathon1 and GAGE datasets showed that SOAPdenovo2 greatly surpasses its predecessor SOAPdenovo and is competitive to other assemblers on both assembly length and accuracy. We also provide an updated assembly version of the 2008 Asian (YH) genome using SOAPdenovo2. Here, the contig and scaffold N50 of the YH genome were ~20.9 kbp and ~22 Mbp, respectively, which is 3-fold and 50-fold longer than the first published version. The genome coverage increased from 81.16% to 93.91%, and memory consumption was ~2/3 lower during the point of largest memory consumption.

## Findings

The increased use of next generation sequencing (NGS) has resulted in an increased growth of the number of *de novo* genome assemblies being carried out using short reads. Although there are several *de novo* assemblers available, there remains room for improvement as shown in recent assembly evaluation projects such as Assemblathon 1 [1] and GAGE [2]. Since the publication of the first version of SOAPdenovo [3], it has been used to assemble many large eukaryotic genomes, but reports have indicated areas that would benefit from updates, including assembly coverage and length [4,5].

SOAPdenovo2, as with SOAPdenovo, is made up of six modules that handle read error correction, *de Bruijn* graph (DBG) construction, contig assembly, paired-end (PE) reads mapping, scaffold construction, and gap closure. The major improvements we have made for in SOAPdenovo2 are: 1) enhancing the error correction algorithm, 2) providing a reduction in memory consumption in DBG constructions, 3) resolving longer repeat regions in contig assembly, 4) increasing assembly length and coverage in scaffolding and 5) improving gap closure. Our data show that SOAPdenovo2 outperforms its predecessor on the majority of the metrics benchmarked in the Assemblathon 1 as well as GAGE; and in addition, was able to substantially improve the original assembly of the Asian (YH) genome [6] that was done using SOAPdenovo.

### Improvements in SOAPdenovo2

Dealing with sequencing error in NGS data is inevitable, especially for genome assembly applications, the outcome of which could be largely affected by even a small amount of sequencing error. Hence it is mandatory to detect and revise these sequencing errors in reads before assembly [2,7]. However, the error correction module in SOAPdenovo was designed for short Illumina reads (35-50 bp), which consumes an excessive amount of computational time and memory on longer reads, for example, over 150 GB memory running for two days using 40-fold 100 bp paired-end Illumina HiSeq 2000 reads. Thus, by a skillful exploitation of data indexing strategies, we redeveloped the module, which supports memory efficient long-*k*-mer error correction and uses a new space *k*-mer scheme to improve the accuracy and sensitivity (see Additional file 1: Supplementary Method 1 and Figures S1-S3). Simulation test shows that the new version runs efficiently and corrects more reads authentically (see Additional file 1: Tables S1 and S2).

In DBG-based large-genome assembly, the graph construction step consumes the largest amount of memory. To reduce this in SOAPdenovo2, we implemented a sparse *de Bruijn* graph method [8] (see Additional file 1: Supplementary Method 2), where reads are cut into *k*-mers and a large number of the linear unique *k*-mers are combined as a group instead of being stored independently.

Another important factor in the success of DBG-based assembly is *k*-mer size selection. Using a large *k*-mer has the advantage of resolving more repeat regions; whereas, use of small *k*-mers is advantageous for assembling low coverage depth and removing sequencing errors. To fully utilize both these advantages, we introduced a multiple *k*-mer strategy [9] in SOAPdenovo2 (see Additional file 1: Supplementary Method 3 and Figure S4). First, we removed sequencing errors using small *k*-mers for graph building, and then we rebuilt the graph using larger *k*-mers iteratively by mapping the reads back to the previous DBG to resolve longer repeats.

Scaffold construction is another area that needs improvement in NGS *de novo* assembly programs [10]. In the original SOAPdenovo, scaffolds were built by utilizing PE reads starting with short insert sizes (~200 bp) followed iteratively to large insert sizes (~10 kbp) [3]. Although this iterative method greatly decreased the complexity of scaffolding and enabled the assembly of larger genomes, there remained many issues that resulted in lower scaffold quality and shorter length. For example, 1) the heterozygous contigs were improperly handled; 2) chimeric scaffolds erroneously built with the smaller insert size PE reads which then hindered the later steps to increase of scaffold length when adding PE reads with larger insert size; and 3) false relationships between contigs without sufficient PE information support were created occasionally. To improve this in SOAPdenovo2, the main changes during the scaffolding stage were as follows: 1) we detected heterozygous contig pairs using contig depth and local contig relationships. Under these conditions, only the contig with higher depth in the heterozygous pairs was kept in scaffold, which reduced the influence of heterozygosity on the scaffolds length; 2) chimeric scaffolds that were built using a smaller insert size library were rectified using information from a larger insert size library, and 3) we developed a topology-based method to reestablish relationships between contigs that had insufficient PE information support (see Additional file 1: Supplementary Method 4 and Figures S5-S7).

Short reads enabled us to reconstruct large vertebrate and plant genomes, but the assembly of repetitive sequences longer than the read length still remain to be tackled. In scaffold construction, contigs with certain distance relationship, but without genotypes amid were connected with wildcards. The GapCloser module was designed to replace these wildcards using the context and PE reads information. In SOAPdenovo2, we have improved the original SOAPdenovo GapCloser module, which assembled sequences iteratively in the gaps to fill large gaps. At each iterative cycle, the previous release of GapCloser considered only the reads that could be aligned in current cycle. This method could potentially make for an incorrect selection at inconsistent locations with insufficient information for distinguishment due to the high similarity between repetitive sequences. For SOAPdenovo2, we developed a new method that considered all reads aligned during previous cycles, which allowed for better resolution of these conflicting bases, and thus improved the accuracy of gap closure. (see Additional file 1: Supplementary Method 5).

### Testing and assessment

**Figure 1:**
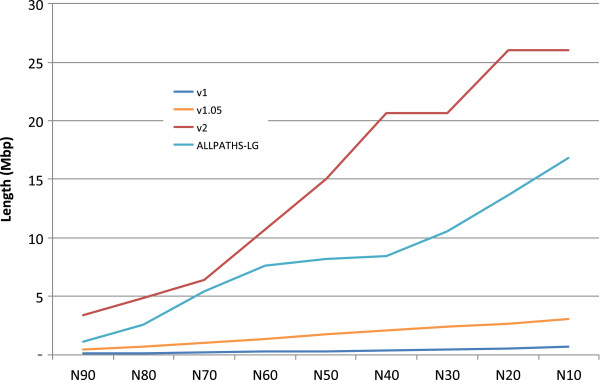
A comparison of the scaffold N10 to N90 between the assemblies based on the Assemblathon 1 dataset.

To test the performance of SOAPdenovo2, we assembled the Assemblathon1 benchmark dataset [11] and evaluated the assembly using the Assemblathon1’s official evaluation pipeline [1]. Our analyses showed that SOAPdenovo2 performed better than the initial release of SOAPdenovo [3] (hereafter referred to as ‘SOAPdenovo1’) and SOAPdenovo v1.05 (hereafter referred to as ‘SOAPdenovo1.05’) used in Assemblathon1. Notably, SOAPdenovo1.05 was developed two years after SOAPdenovo1 for the Assemblathon1 and has never been formally released. It included partial improvements and new features from SOAPdenovo2, including the new contig and scaffold construction improvements, but without the new error correction and gap closure modules. Compared with the results of SOAPdenovo1, the new scaffold N50 was nearly an order of magnitude longer and the accuracy was higher due to the reduction of structural error by 90.12%, substitution error by 92.13%, and copy number error by 69.47% (Table 1, Figure 1). We also compared our results with that of ALLPATHS-LG [5], and SOAPdenovo2 produced contig N50 and scaffold N50 that were approximately 1.53 and 1.84-times longer. The SOAPdenovo2 assembly also had a much lower amount of copy number errors, but did have more substitution errors [1]. The lower substitution error in ALLPATHS-LG is likely because it includes a step analogous to “editing the assembly” to eliminate ambiguity, but it does so at the expense of more computational consumption. Improvements of SOAPdenovo2 have also been observed in assembling GAGE [8] dataset (see Additional file 1: Supplementary Method 6 and Tables 2 and 3). As shown in Tables 2 and 3, the correct assembly length of SOAPdenovo2 increased by approximately 3 to 80-fold comparing with that of SOAPdenovo1. Worth mentioning, there are only two levels of insert size for *Staphylococcus aureus* and *Rhodobacter Sphaeroides*, the setting of which is optimal for ALLPATHS-LG, but mismatches with the requirement of SOAPdenovo2 to come up with an optimal assembly (see Additional file 1: Supplementary Method 4); thus, the results of GAGE might not be able to illustrate the power of SOAPdenovo2, especially for the scaffolding part.

**Table 1:** Evaluation of Assemblathon1 dataset assemblies

	**Contig N50**	**Contig path NG50**	**Scaffold N50**	**Scaffold path NG50**	**Number of Structural Error**	**Substitution Error rate**	**Copy Number Error rate**	**Genome coverage (%)**	**Memory (G)**	**Run time (h)**
V1	207,783	13,357	329,384	13,539	14,306	5.40E-05	9.14E-03	98.8	46	7
V1.05*	343,889	82,264	1,684,436	116,651	1,878	1.20E-05	6.75E-03	98.8	20	8
V2.0	357,238	111,365	15,077,357	170,432	1,414	4.25E-06	2.79E-03	98.8	20	10^§^
ALLPATHS-LG*	163,633	72,480	8,185,650	210,649	1,244	2.92E-06	6.71E-02	98.3	100	12

Contig and scaffold path NG50 were defined in Assemblathon1 [1].

^*^SOAPdenovo v1.05 and ALLPATHS-LG’s evaluation result data were from [1].

^§^Time spent on filtering contamination was not included.

**Table 2:** **Assemblies of *S. aureus *** **and *R. sphaeroides***

**Species**	**Version**	**Contigs**				**Scaffolds**			
		**Number**	**N50 (kb)**	**Errors**	**N50 corrected(kb)**	**Number**	**N50 (kb)**	**Errors**	**N50 corrected (kb)**
*S. aureus*	SOAPdenovo1	79	148.6	156	23	49	342	0	342
	SOAPdenovo2	80	98.6	25	71.5	38	1,086	2	1,078
	ALLPATHS-LG*	37	149.7	13	117.6	10	1,477	1	1,093
*R. sphaeroides*	SOAPdenovo1	2,242	3.5	392	2.8	956	105	18	70
	SOAPdenovo2	721	18	106	14.1	333	2,549	4	2,540
	ALLPATHS-LG*	190	41.9	31	36.7	32	3,191	0	3,310

All datasets were downloaded from http://gage.cbcb.umd.edu/data/.

*ALLPATHS-LG was using the latest version 42807.

**Table 3:** **Assemblies of *Bombus Impatiens***

**Assembler**	**Contigs**			**Scaffolds**		
	**Number**	**N50 (kb)**	**E-size (kb)**	**Number**	**N50 (kb)**	**E-size (kb)**
SOAPdenovo1	64,361	7.9	10.4	52,041	12	25
SOAPdenovo2	12,550	75.7	91.1	5,084	1,352	1,596
ALLPATHS-LG*	-	-	-	-	-	-

*The published ALLPATHS-LG could not be used to assemble this genome because it requires at least one library with overlapping paired-end reads.

We also used SOAPdenovo2 to reassemble and update the previously assembled YH Asian Genome [12]. The previous assembly was done using SOAPdenovo1 [3], but in addition it was also limited by the very short read lengths (~35 bp) that were the standard output of Illumina Genome Analyzers (GAIIx) at that time and by the insert sizes available (maximum size is 10 kb). To provide an updated assembly with the new program, we generated a new set of PE 100 bp-long reads with an insert size ranging from 180 bp to 40 kbp using the Illumina HiSeq 2000 [13] (see Additional file 1: Table S3). These new data were put through both the SOAPdenovo1 and SOAPdenovo2 pipelines. To test out the performance of each new feature in SOAPdenovo2, we also assembled the genome with or without the multi *k*-mers and sparse DBG modules.

**Figure 2:**
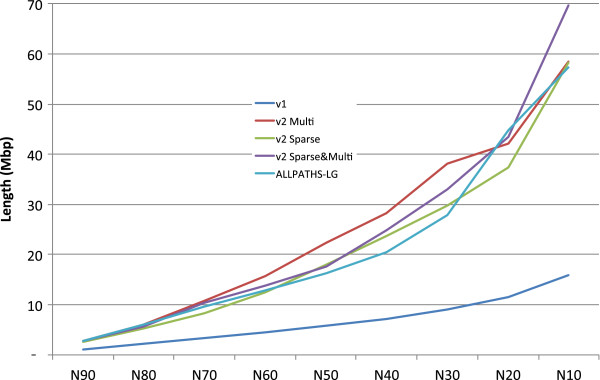
A comparison of the scaffold N10 to N90 between the assemblies based on the new YH sequencing data.

As shown in Table 4 and Figure 2, using the new data, we found that the Contig N50 and Scaffold N50 of SOAPdenovo2 were, respectively, 1.64 and 3.84-times longer than SOAPdenovo1. The result is also 3-fold and 50-fold longer than the first YH genome version. Notably, by using sparse DBG, the memory consumption for graph construction decreased dramatically, but the N50 contig and N50 scaffold dropped. This is due to the shorter *k*-mer length required by sparse DBG’s design to acquire higher *k*-mer depth, which in turn disabled some repetitive sequences from being solved (see Additional file 1: Supplementary Method 2). By using larger *k*-mer length, ALLPATHS-LG outperformed SOAPdenovo2 on contig N50 by 1.49-times, but for scaffold N50, SOAPdenovo2 is 6 Mbp (1.37-times) longer. SOAPdenovo2 covered the reference genome 5.38% more and ran 3.36-times faster on the same machine than ALLPATHS-LG. To confirm the contribution of new algorithms, we evaluated both the YH genome assembled by SOAPdenovo1 and SOAPdenovo2 respectively by aligning them to the NCBI human reference genome hg19 [14]. We obtained a reference coverage increase from 81.2% to 93.9%, and we found that approximately 95.9% of the newly assembled regions were repetitive sequences. The increased reference coverage is mainly due to the improved SOAPdenovo2, not to the newly sequencing data.

**Table 4:** Summary of YH dataset assemblies

**Data and Program**	**Version**	***k*-mer**	**Scaffold total length (bp)**	**Scaffold N50 (bp)**	**Contig total length (bp)**	**Contig N50 (bp)**	**Coverage**	**Time (h)**	**Peak Memory at Graph Construction (G)**
SOAPdenovo YH old data	v1	25	2,837,024,602	455,380	2,327,931,678	4,933	80.51%	48^^^	140
SOAPdenovo YH new data	v1	31	2,901,125,426	5,806,495	2,661,982,498	12,709	81.16%	58^^^	107
	v2 Multi-*k*-mer	45-61	2,905,148,690	22,297,138	2,799,723,051	20,926	93.91%	74^^^	155
	v2 Sparse	35	2,874,598,201	18,033,622	2,767,141,367	18,856	93.17%	78^^^	35
	v2 Sparse & Multi-*k*-mer	35-49	2,888,094,847	17,576,272	2,776,209,134	18,960	93.20%	81^^^	35
ALLPATHS-LG^§^ YH new data	42807	96	2,809,141,261	16,195,684	2,600,792,533	31,101	88.53%	249^*^	343

To be consistence with the result of ALLPATHS-LG, contigs and scaffolds shorter than 1 kb were filtered for SOAPdenovo assemblies.

§ Without ‘FixLocal’ due to the module failure (see Additional file 1: Supplementary Method 7).

^ Time consumption including SOAPdenovo’s error correction, assembly and gap closure modules.

* Time consumption including ALLPATHS-LG’s preparation and assembly modules.

A previous report had indicated that most of the segmental duplications (SD) were lost in the earlier published version of the YH [4]. To investigate the SD coverage of new version YH genome sequences, we aligned the contigs of the first version and the new version to 134 Mb of published human SD sequences [15] and found that up to 99% of the published SD sequences were now sufficiently represented (≥ 90% of each sequence) in the updated assembly, while only 21.5% were represented in the earlier version (see Additional file 1: Table S4). The rate of SD sequences that appeared more than once with sufficient coverage for each copy was increased from 0.02% to 52.6% in the updated version. The assembly of fragmented genes (noted in [4]) was also improved (see Additional file 1: Table S5). For example, average coverage of gene *GRM5* increased from 90% to 96% and the number of fragments decreased from 162 to 4.

The work here demonstrates that SOAPdenovo2 is greatly improved over the initial version and specifically in areas that have been highlighted as problems in the currently available short-read *de novo* assembly programs. It thus provides an effective solution for carrying out *de novo* genome assembly especially for eukaryotic genomes. We have also been able to provide a much better quality version of the previously assembled YH genome [13], which will serve as an excellent reference genome for use in Chinese population studies, as well as for general human genome studies. SOAPdenovo2 has been successfully deployed in public computing clouds including TianHe series supercomputer and Amazon EC2.

## Availability and requirements

• Project name: SOAPdenovo2

• Project home page and forum: http://soapdenovo2.sourceforge.net/

• Operating system(s): Unix, Linux, Mac

• Programming language: C, C++

• Other requirements: GCC version ≥ 4.4.5

• License: GNU General Public License version 3.0 (GPLv3)

• Any restrictions to use by non-academics: none

Contact: bgi-soap@googlegroups.com

## Availability of supporting data

The raw reads from the YH genome generated in this work are available from the BGI website [16], the EBI short read archive with study accession [EMBL:ERP001652], and also from the *GigaScience* database [6]. The updated assembly is also available at *GigaScience*[13]. In order to facilitate readers to repeat the experiments, the tools and configured packages including commands and necessary utilities are available from our FTP server ftp://public.genomics.org.cn/BGI/SOAPdenovo2, and are also being made available from the *GigaScience* database [17].

## Abbreviations

bp: Base pair; DBG: *de Bruijn* graph; PE: Paired end; SD: Segmental duplication; YH: Asian genome.

## Competing interests

The authors declare that they have no competing interests.

## Authors’ contributions

RL, BL, YX and ZL contributed equally to this work. Ju W, Ji W, HY, TL, Yi L and RL managed the project. RL, BL, YX and ZL led the design. RL, WH, JY, GH, YC, QP, YL, JT, GW, HZ, YS, Yo L, CY, DC, SY, XZ, SP, XL and GL implemented and tested the software. BW, Ya L, CH performed sequencing. RL and BL wrote the paper. All authors read and approved the final manuscript.

## Supplementary Material

Additional file 1 **Supplementary Method 1 P2 Improvement of Error Correction module in SOAPdenovo2.** Supplementary Method 2 P5 Construction of sparse *de Bruijn* graph in SOAPdenovo2. Supplementary Method 3 P5 Improvement of contig building in SOAPdenovo2. Supplementary Method 4 P7 Improvement of the Scaffolding module in SOAPdenovo2. Supplementary Method 5 P8 Improvement of the GapCloser module in SOAPdenovo2. Supplementary Method 6 P9 Evaluating the GAGE dataset. Supplementary Method 7 P9 Updating the YH genome assembly. Supplementary Method 8 P10 Evaluation of the YH genome. Supplementary Method 9 P10 Machine used. **Table S1.** P11 Error correction results of simulated *Arabidopsis thaliana* reads. **Table S2.** P11 Computational resources consumption of error correction programs. **Table S3.** P11 Summary of the production of the new YH dataset. **Table S4.** P11 Coverage of published SD sequences of the YH genome. **Table S5.** P12 Coverage and fragments on repetitive genes of the YH genomes. **Table S6.** P12 The parameters used in SOAPdenovo2’s pipeline for YH assembly. **Figure S1.** P14 An illustration of co-op between Consecutive *k*-mer and Space *k*-mer. **Figure S2.** P14 An example of base correction by FAST approach. **Figure S3.** P15 An illustration of base correction by DEEP approach. **Figure S4.** P16 The workflow of building sparse DBG in SOAPdenovo2. **Figure S5.** P16 The contig type distribution of Human X Chromosome and *Arabidopsis thaliana.***Figure S6.** P17 A theoretical topological structure of heterozygous contig pairs. **Figure S7.** P18 The detection and rectification of chimeric scaffolds. Click here for file
