# Erratum: SOAPdenovo2: an empirically improved memory-efficient short-read de novo assembler

**DOI:** 10.1186/s13742-015-0069-2

**Published:** 2015-07-08

**Authors:** Ruibang Luo, Binghang Liu, Yinlong Xie, Zhenyu Li, Weihua Huang, Jianying Yuan, Guangzhu He, Yanxiang Chen, Qi Pan, Yunjie Liu, Jingbo Tang, Gengxiong Wu, Hao Zhang, Yujian Shi, Yong Liu, Chang Yu, Bo Wang, Yao Lu, Changlei Han, David W. Cheung, Siu-Ming Yiu, Shaoliang Peng, Zhu Xiaoqian, Guangming Liu, Xiangke Liao, Yingrui Li, Huanming Yang, Jian Wang, Tak-Wah Lam, Jun Wang

**Affiliations:** 1 BGI HK Research Institute, 16 Dai Fu Street, Tai Po, Industrial Estate, Tai Po, Hong Kong; 2 HKU-BGI Bioinformatics Algorithms and Core Technology Research Laboratory & Department of Computer Science, University of Hong Kong, Pok Fu Lam, Hong Kong; 3 School of Bioscience and Bioengineering, South China University of Technology, Guangzhou, 510006 China; 4 School of Computer Science, National University of Defense Technology, No.47, Yanwachi street, Kaifu District, Changsha, 410073 Hunan China

## Erratum

After the publication of ‘SOAPdenovo2: an empirically improved memory-efficient short-read de novo assembler. *GigaScience* 2012 1:18’ [1] this paper was used as case study by the ISA, Research Object and Nanopublication communities to use their data models to try to quantitatively assess the reproducibility of published research. While the supporting data and overall results were found to be accurate, a number of semantic problems were detected in a few points of interpretation, and in order to ensure the accuracy of the scientific we now outline these in this correction article.

1. While there are huge improvements to the quality of the resulting assemblies, other than the tables it was not stressed in the text that the speed of SOAPdenovo2 can be slightly slower than SOAPdenovo v1.
2. In the testing an assessment section (page 3), based on the correct results in table 1, where we say the scaffold N50 metric is an order of magnitude longer from SOAPdenovo2 versus SOAPdenovo1, this was actually 45 times longer.
3. Also in the testing an assessment section, based on the correct results in table 1, where we say SOAPdenovo2 produced a contig N50 1.53 times longer than ALLPATHS-LG, this should be 2.18 times longer.
4. Finally in this section, where we say the correct assembly length produced by SOAPdenovo2 was 3–80 fold longer than SOAPdenovo1, this should be 3–64 fold longer.

To see more about how these errors were detected see the case study article [2]. To access all the supporting materials, ISA metadata and pipelines implemented in Galaxy to test these results on your own, please see the supporting entry in GigaDB [3].
