# Supplementary material for: SOAPdenovo2: an empirically improved memory-efficient short-read de novo assembler
Source: Gigascience. 2012 Dec 27;1:18. doi: 10.1186/2047-217X-1-18 (PMC3626529; doi:10.1186/2047-217X-1-18)
Supplement: Additional file 1 — Supplementary Method 1 P2 Improvement of Error Correction module in SOAPdenovo2. Supplementary Method 2 P5 Construction of sparse de Bruijn graph in SOAPdenovo2. Supplementary Method 3 P5 Improvement of contig building in SOAPdenovo2. Supplementary Method 4 P7 Improvement of the Scaffolding module in SOAPdenovo2. Supplementary Method 5 P8 Improvement of the GapCloser module in SOAPdenovo2. Supplementary Method 6 P9 Evaluating the GAGE dataset. Supplementary Method 7 P9 Updating the YH genome assembly. Supplementary Method 8 P10 Evaluation of the YH genome. Supplementary Method 9 P10 Machine used. Table S1. P11 Error correction results of simulated Arabidopsis thaliana reads. Table S2. P11 Computational resources consumption of error correction programs. Table S3. P11 Summary of the production of the new YH dataset. Table S4. P11 Coverage of published SD sequences of the YH genome. Table S5. P12 Coverage and fragments on repetitive genes of the YH genomes. Table S6. P12 The parameters used in SOAPdenovo2’s pipeline for YH assembly. Figure S1. P14 An illustration of co-op between Consecutive k-mer and Space k-mer. Figure S2. P14 An example of base correction by FAST approach. Figure S3. P15 An illustration of base correction by DEEP approach. Figure S4. P16 The workflow of building sparse DBG in SOAPdenovo2. Figure S5. P16 The contig type distribution of Human X Chromosome and Arabidopsis thaliana.Figure S6. P17 A theoretical topological structure of heterozygous contig pairs. Figure S7. P18 The detection and rectification of chimeric scaffolds. [file 2047-217X-1-18-S1.docx]

Additional file 1

SOAPdenovo2: An empirically improved memory-efficient short-read
*de novo* assembler

| Supplementary Method 1 | P2 | Improvement of Error Correction module in SOAPdenovo2 |
| --- | --- | --- |
| Supplementary Method 2 | P5 | Construction of sparse *de Bruijn* graph in SOAPdenovo2 |
| Supplementary Method 3 | P5 | Improvement of contig building in SOAPdenovo2 |
| Supplementary Method 4 | P7 | Improvement of the Scaffolding module in SOAPdenovo2 |
| Supplementary Method 5 | P8 | Improvement of the GapCloser module in SOAPdenovo2 |
| Supplementary Method 6 | P9 | Evaluating the GAGE dataset |
| Supplementary Method 7 | P9 | Updating the YH genome assembly |
| Supplementary Method 8 | P10 | Evaluation of the YH genome |
| Supplementary Method 9 | P10 | Machine used |
| Supplementary Table 1 | P11 | Error correction results of simulated *Arabidopsis thaliana* reads |
| Supplementary Table 2 | P11 | Computational resources consumption of error correction programs |
| Supplementary Table 3 | P11 | Summary of the production of the new YH dataset |
| Supplementary Table 4 | P11 | Coverage of published SD sequences of the YH genome |
| Supplementary Table 5 | P12 | Coverage and fragments on repetitive genes of the YH genomes |
| Supplementary Table 6 | P12 | The parameters used in SOAPdenovo2’s pipeline for YH assembly |
| Supplementary Figure 1 | P14 | An illustration of co-op between Consecutive *k*-mer and Space *k*-mer |
| Supplementary Figure 2 | P14 | An example of base correction by FAST approach |
| Supplementary Figure 3 | P15 | An illustration of base correction by DEEP approach |
| Supplementary Figure 4 | P16 | The workflow of building sparse DBG in SOAPdenovo2 |
| Supplementary Figure 5 | P16 | The contig type distribution of Human X Chromosome and *Arabidopsis thaliana* |
| Supplementary Figure 6 | P17 | A theoretical topological structure of heterozygous contig pairs |
| Supplementary Figure 7 | P18 | The detection and rectification of chimeric scaffolds |
| References | P19 |  |

### Supplementary methods

1. **Improvement of Error Correction module in SOAPdenovo2**

SOAPdenovo is based on the *de Bruijn* graph data structure, which uses nodes to represent all possible *k*-mers (a *k*-mer is a substring of read of length *k*), and edges to represent perfect overlap of heads and tails of length *k*-1. However, in *de Bruijn* graphs, each base error in a read is supposed to introduce up to *k* false nodes. These false nodes waste excessive amounts of computational time and memory, and since each false node would have a chance linking to other authenticate nodes, it is possible to induce fake path convergence. Meanwhile, with the rapid development of sequencing technology, larger *k* sizes have been adopted to take the advantage of longer reads produced by various platforms, this in turn introduces much more false nodes that would exceed the computational capability, which hinders us from assembling large vertebrate genomes using the latest sequencing technology. Thus, detecting and fixing base errors in reads in advance of assembly will lead to higher assembly efficiency and quality.

We have improved the error correction module to SOAPec-2.0. The algorithm is based on k-mer frequency spectrums (KFS), but the algorithm is quite different from other KFS tools. We therefore describe the new algorithm used in SOAPec-2.0 here.

SOAPec-2.0 consists of four mandatory stages and one optional stage according to the input: (1) Construct the KFS; (2) Examine and select reads with possible error for correction; (3) Fix sparsely distributed erroneous bases with a fast voting algorithm called “FAST”; (4) Fix adjacent or nearby bases as well as the errors at the edges of reads that failed to be corrected in stage 3 using a more complicated but slower algorithm called “DEEP”; (5) Optional trim (or discard entirely) the fixed reads that remain erroneous. The details are described as follows:

1. Construct the *KFS.*

In error correction, *k*-mer size should be appropriately chosen based on the genome size in order not to confuse error *k*-mers with correct *k*-mers. For example, when the genome size is 3G without repeat, the maximum species number of correct *k*-mers of arbitrary length *k* is 3G-*k*, and considering the two strands, we need two times the *k*-mer entries (6G) to store the *k*-mer frequency. The species number of false *k*-mers, which were caused by random sequencing errors, will be much higher than the correct *k*-mers. In order not to confuse correct *k*-mers with the large number of false *k*-mer in residence, we recommend to set the *k*-mer space defined by *k* at least 10 times larger than the species number of correct *k*-mers. The following formula helps users in choosing the *k*-mer size: $4^{k}\geq GenomeSize\times2\times10$. For human genome specifically (3G in size), the *k*-mer space required should be 4 *^k^* ≥ 60G, so we would suggest use of a *k*-mer size equal to or larger than 18.

In our algorithm, we define two kinds of *k*-mers: Consecutive *k*-mer and Space *k*-mer (Figure S1). In a read *r*, a Consecutive *k*-mer is a substring *r*[*i, i+1, …, i+k*] start from *i* with *k* bp in length. Space *k*-mer has one gap with length *s* inside the substring *r*, a space *k*-mer might be *r*[*i, i+*1*, …, i+k/*2*, i+s+k/*2*, …, i+s+k*]. Only the Consecutive *k*-mer was used in SOAPec-1.0. SOAPec-2.0 utilizes both the Consecutive *k*-mer and the Space *k*-mer simultaneously.

To construct the KFS for Consecutive *k*-mer and Space *k*-mer, we provide two approaches. The first approach using index table requires at most 4*^k^* bytes of memory; with the frequency each *k*-mer occupies a byte that can support frequency up to 255. Another approach stores *k*-mers and their frequencies in a hash table data structure, the memory requirement of which is based on the species number of *k*-mer in dataset (including correct and false *k*-mers). The first approach notably consumes memory stably, while the second approach is depends on the data quality, hence undeterministic. With a *k*-mer size ≤17 bp, the first approach is recommended because the program speed is faster and consumes less memory. In contrast, the second approach is recommended when *k*-mer size is larger than 17 bp.

1. Examine and select reads with possible errors for correction.

Before error correction, we firstly need to import the *k*-mer frequency tables into the memory. Here we divide the *k*-mers into two categories, low frequency *k*-mer (0) and high frequency *k*-mer (1). Only a bit is used to keep the type of each *k*-mer. So, in order to keep a 17-mer table, we need merely 2G in memory, which reduced the memory consumption compare with SOAPec-1.0.

For each read input, we detect and divide it into a set of consecutive false *k*-mer blocks and authentic *k*-mer blocks, and store these information into a vector of a data structure, which has three elements including the block starting position, ending position and its status (low frequency block or high-frequency block). Reads with low frequency *k*-mer blocks are considered with possible errors and will be passed to next correction stage.

1. Correct trivial errors by “FAST” approach.

Using *k*-mer of length *k*, a single sequencing error with no second error in *k* bp flanking region, occurs at position *s* of a read with length *x*, will ideally transform up to *min(s, k, x-s)* false *k*-mers. The aim of our “FAST” approach is to transform *min(s, k, x-s)* false *k*-mers to authentic *k*-mers in *K_c_*. To achieve this, a voting algorithm is applied to correct the error base that result in these false *k*-mers. The algorithm substitute the error base by iterating all possible bases and then check the authenticity of new generated *k*-mers corresponding to the error base. An error base is marked as corrected if one and ONLY one substitution can transform all false *k*-mers to authentic. The “FAST” algorithm is illustrated in Figure S2.

The pseudo code of the “FAST” algorithm is shown as follows:

*//Start*

*p* <- 0;

for *b* in A,G,C,T{

*c* <- 0;

*r*[*s* + *k_c_* - 1] = *b*;

for(*i* = 0; *i* < *k_c_*; ++*i*)

{

*pos* <- *s* + *i*;

*k-mer* := a copy of r[*pos*, …, *pos + k_c_* - 1];

if(*k-mer* belongs to *K_c_*){

*c* <- *c* + 1;

}

}

if(*c* == *k_c_*){

*p* <- *p* + 1;

}

}

if(*p* == 1)

accept the change;

//End

1. Correct complicated errors by “DEEP” approach.

While “FAST” aims to modify trivial types of errors rapidly, the “DEEP” approach aims to correct errors failed by “FAST” and these errors may share following characteristics: 1) with adjacent or nearby error; 2) located in both edges of a read and 3) corresponding segment is a subset of repetitive sequences. These characteristics avoid the errors from being corrected by voting a single base alternation; and hence, have to be corrected by referring to context correction. In the “DEEP” algorithm, a substring prefixing the forefront of a possible base error, with all including k-mers are authentic, is defined as a head node to be extended in a branch and bound tree. All possible extension paths will be appended to the head node until the accumulated base change c is exceeding the user defined maximum *c_max_*. A path will be finally selected if the path is the ONLY one to have the lowest *c*. Corresponding error bases will be modified by traversing back from the top level child of the selected path (Figure S3).

The pseudo code of the “DEEP” algorithm is shown as follows:

//Start

push *e*(*base* = null, *change* = 0) into N_0_;

for(*i* = 0; *i* < *k*; ++*i*)

{

foreach element *e* in *Ni*{

*pos* <- *s* + *i*;

for *b* in A,G,C,T{

*k-mer_consecutive_* := a copy of *r*[*pos*, …, *pos* + *k_c_* - 2] + *b*;

*k-mer_space_* := a copy of *r*[*pos*, …, *pos* + *l_s_* - 2] + *b*;

*c* <- *e*.*change*;

if(*e*.*base* != *r*[*i*]){

*c* <- *c* + 1;

}

if(*c* <= *c_max_*){

if(*k-mer_consecutive_* belongs to *K_c_*){

if(*k-mer_space_* belongs to *K_s_*){

push *e*(*base = b*, *change = c*) into *N_i+1_*;

}

}

}

}

}

}

foreach element *e* in *N_k_*{

if (*e.change* != 0){

if (*e.change* is the ONLY minimum in *N_k_*){

accept the changes;

}

}

}

//End

1. Optional trim (or discard entirely) the fixed reads that remain as errors.

This stage attempts to find the longest substring of the read, in which *k*-mers are all authentic. Manually disabling, trimming or discarding reads will let uncorrected error bases coexist with fixed bases in reads, which passes the correction workload to downstream genome assemblers, which adopt consensus context for more extensive filtering or correction.

We simulated 30-fold 100bp pair-end reads from *Arabidopsis thaliana* with 0.5% base error by pIRS [1]. Then we used SOAPec-1.0, SOAPec-2.0, SOAPec-v2.0-DuoKmer and Quake [2] to correct these simulated data. The corrected results were shown in Table S1 and Table S2. It is worth mentioning that there are more reads remained after the correction of SOAPec-2.0 than Quake, while sensitivities are the same. Almost all the metrics of SOAPec-2.0 including false positive (FP), false negative (FN) and true positive (TP) are better than SOAPec-1.0 and Quake. It is necessary to mention that the correction performance of SOAPec-v2.0-DuoKmer is better than all other three programs. Compared with SOAPec-1.0, the memory consumption of SOAPec-2.0 reduced from 30 GB to less than 4 GB during correction and the time consumption decreased by eight times.

1. **Construction of sparse** ***de Bruijn* graph in SOAPdenovo2**

A key problem of *de Bruijn* graph (DBG) based genome assemblers is the large computational memory requirement for graph construction. A year ago, Chengxi Ye developed a method to construct so called ‘sparse k-mer graph’[[3](#_ENREF_3)], or ‘sparse DBG’ in our case. The method simply stores only one out of every *g* (*g* < *k*) *k*-mers, attempting to sub-sample as evenly across the original *de Bruijn* graph as possible. The size of the de Bruijn graph is reduced by a factor of approximately *g* in theory. In our implementation, it reduces the memory consumption by 2-5 times in graph construction step. Different from Ye’s design (SparseAssembler), our algorithm could be processed in parallel and also fits the sophisticated contig construction algorithms in SOAPdenovo well. A workflow of the sparse DBG construction in SOAPdenovo2 is shown in Figure S4. Notably, the graph construction procedure is order-dependent, i.e. different input reads ordering would results in different graph structure. While multi-threading functionality alter the sequence of input reads, the assembly output would be slightly different with different thread numbers set.

The sparse DBG module requires the user to input an estimated genome size to guide the memory allocation. However, the constraint of the data structure makes the module unable to provide a unique result with different estimated genome size. Different size of memory allocation alters the starting point of the graph traversal. We will fix the problem in the near future.

The sparse DBG method also requires shorter k-mer length compared to the full graph method. The ability of encoding overlaps between reads within the sparse k-mer graph is between using that of de Bruijn graphs constructed with k-mer sizes between k and (k+g). For more details please refer to [[3](#_ENREF_3)].

Due to the limitations of sparse DBG, we suggest the use of the full graph method on small genomes and repetitive genomes and only use sparse DBG when the memory is limited.

1. **Improvement of contig building in SOAPdenovo2**

For DBG-based assembly, it is not trivial and intuitive to choose a proper *k*-mer size for an optimized contig result due to the reason that, the final contig length for whole genome *de novo* assembly is related to many factors, including *k*-mer size, sequencing depth, sequencing error, repetitive patterns distribution along the genome and the heterozygosity of the sequenced sample.

Here we adopt the definition of *k-*mer depth from a previous study [4]. To obtain longer contigs, we should firstly make sure the *k*-mer depth is substantial to indicate authentic transitions between adjacent *k*-mers. As shown in the previous study and also Liu *et al.* [5], the *k*-mer depth is a product of *k*-mer size, read length and sequencing depth. Larger *k* will reduce the *k*-mer depth and decrease the contig length in turn; so, sufficient *k*-mer depth should be guaranteed in the first place.

The determination of *k* should also consider the repetitive patterns distribution along the genome. As shown in Figure S5, reads simulated from the Human X Chromosome and *Arabidopsis* with the same parameters were assembled using SOAPdenovo with identical *k*-mer size. The contigs were then categorized into four types by being mapped to the reference genome. The four types are: ‘error contigs’ (contigs containing sequencing error), ‘unique contigs’ (contigs that could be aligned to the reference genome with unique position), ‘similar contigs’ (contigs that could be fully aligned to the reference at a position, and to other positions with identity larger than 95%), and ‘repeat contigs’ (contigs that could be fully aligned to the reference genome with at least two positions). Different distribution of the number of contig types is a result of the lengths of assembled contigs and the complexity of the reference genome. For genomes with more short repetitive patterns such as *Arabidospsis*, we suggest large *k*-mer sizes that can handle these patterns effectively are adopted in assembly.

*k* size determination is also related to sequencing error and the heterozygosity of the sequenced genome. High sequencing error or heterozygosity will drag down the contig length. Consequently, large *k*-mer size could make the assembly even worse with high heterozygosity as a result of diverging haploids. For a complex genome, it is difficult to determine the optimal *k*-mer size based on theory.

As mentioned above, large *k*-mer size might solve the problem of short repeats, which will increase the quality contig assembly if sequencing depth permit; small *k*-mer size will increase *k*-mer depth and reduce the side-effect of sequencing error and heterozygosity. To fully utilize the advantages of small *k*-mer size and large *k*-mer size, a multiple *k*-mers strategy has been studied and implemented firstly by Yu Peng [6]. The basic idea of this method is to, firstly, use smaller *k*-mers to distinguish sequencing errors and merge highly heterozygous regions. Then, larger *k*-mers are used to converge small repeats. The multi-*k*-mer algorithm implemented in SOAPdenovo2 is shown as below in pseudo code:

//Start

*k* <- *k*_min_ (*k*_min_ is set at graph construction ‘pregraph’ step);

Construct initial *de Bruijn* graph with *k*_min_;

Remove low depth *k*-mers and cut tips;

Merge bubbles of the de Bruijn graph;

Repeat {

*k* <- *k* + 1;

Get contig graph *H_k_* from previous loop or construct from *de Bruijn* graph;

Map reads to *H_k_* and remove the reads already represented in the graph;

Construct H_k+1_ graph base on *H_k_* graph and the remaining reads with *k*;

Remove low depth edges and weak edges in *H_k_*;

} Stop if *k* >= *k_max_*(k_max_ = k set in contig step(-m));

Cut tips and merge bubbles;

Output all contigs;

//End

The multi-*k*-mer strategy will increase the assembly time consumption, but longer contigs could be obtained using this method.

1. **Improvement of the Scaffolding module in SOAPdenovo2**

Contigs intrinsically break at the repetitive sequences that could not be solved with certain *k*-mer length, thus scaffolding based on paired-end reads information is necessary. As mentioned in the first version of SOAPdenovo [7], two ideas were implemented to facilitate the scaffolding procedure. 1) Contigs shorter than a threshold and ‘likely repetitive contigs’ are masked before scaffolding, thus simplifying the contig graph, and 2) build scaffolds hierarchically traversing from short insert size to large insert sizes. Although these two ideas greatly decreased the complexity of scaffolding and enabled the assembly of larger vertebrate genomes, there are still several problems that cause low scaffold quality and short scaffold length. Three main problems have been scrutinized and addressed in SOAPdenovo2, these details being as follows:

Firstly, the heterozygous contig pairs were improperly handled in SOAPdenovo. Homologs that contain substantial amounts of SNPs and short indels might be separately assembled into two contigs (contig pairs) using DBG. These contig pairs will be located to the same or almost the same (diversified by the distribution of a insert size) position in a scaffold because of similar relation to other adjacent contigs. However, there exists no paired-end relationship between the contig pairs, which may cause a conflict that will stop the extension of the scaffold as shown in Figure S6.

In SOAPdenovo2, heterozygous contig pairs are recognized by utilizing the information of contig depth and the locality of contig. The recognized heterozygous contig pairs should obey the following rules: 1) the similarity between contigs should be high enough, for example, ≥ 95%; 2) the depth of both contigs should be near half of the average depth or all contigs, complying Poisson distribution; 3) the two contigs should be located adjacently in a scaffold and have no relationship to each other inferred by paired-end reads information. The normal contigs neighboring the heterozygous regions, if they exist, could be connected to both of the heterozygous contig pairs (H1 and H2). Only the contig with relatively higher depth in a heterozygous contig pair were kept for scaffolding. The method reduces the influence of genome heterozygosity on final scaffold length. All heterozygous contig pairs were outputted to a file to facilitate further analysis. However, the trade-off of this method is that it might incorrectly remove paralogous contigs. This problem could be relieved by a gap-filling procedure while the removed copy of paralogous contigs would be represented by gaps during scaffolding.

The second is the chimeric scaffold problem. Since SOAPdenovo uses the paired-end reads of shorter insert size in the first place, chimeric scaffolds, comprising of contigs far away from each other along the genome, can be assembled together incorrectly. This is caused either by the contigs containing repetitive regions longer than the insert size or by the lack of sufficient links at the divergences in the contig graph. Chimeric scaffolds were erroneously created during the utilization of small insert size paired-end reads and might hinder the increase of scaffold length when adding paired-end reads with larger insert sizes. In SOAPdenovo2, chimeric scaffolds incorrectly built are examined and rectified before further extending using the libraries with a larger insert size.

In detail, when importing paired-end information with a larger insert size, we recognize these chimeric scaffolds and revise them before using these new paired-end relationships to extend scaffolds. The chimeric scaffolds usually have the following characteristics: 1) contigs on both sides of the chimera-causing contig (with long repetitive sequencing, long than the insert-size) would have few or even no links to other contigs supported by the paired-end reads; 2) contigs on the left side of the chimeric-causing contig have links to some already well-extended scaffolds, while contigs on the right side have links to other scaffolds also well extended.

Scaffolds complying with the above two characteristics would be cut off at the boundary of the chimera-causing contig. This enables the two shorter scaffolds to connect to other scaffolds or contigs correctly. There are two advantages using this strategy. Firstly, it detects and breaks chimeric scaffolds much earlier with multiple levels of insert size, such that the chimeric errors will not be inherited and hinder the scaffold from extension. Secondly, it avoids improper masking of contigs in chimeric scaffolds, and hence there remains more useful contig information for scaffold construction.

The third problem is the incorrect relationships created between contigs. Relationships between contigs without sufficient explicit paired-end information were often treated improperly in SOAPdenovo1. In SOAPdenovo2, we developed a topology-based method to establish and scrutinize relationships between contigs that had insufficient explicit paired-end information. There are four reasons to have insufficient relationships between two adjacent contigs: 1) sequencing depth is insufficient; 2) the two contigs should not be adjacent to each other, but mistakenly brought together by repeat contigs; 3) the two contigs are disordered and should be exchanged, causing by the deviation of the insert size; and 4) the two contigs are homologs (i.e., a heterozygous contig pair). To cope with the problem, we reestablished the relationships between two contigs when fulfilling the following criteria: 1) the two contigs are not a heterozygous contig pair,; 2) the deviation of insert size covers the reverse relationship of two contigs; 3) the two contigs are probably adjacent to each other supported by other contigs using alignment.

1. **Improvement of the GapCloser module in SOAPdenovo2**

In each scaffold, the regions between contigs with approximate base count, but without genotypes are named as gaps and represented by character ‘N’. Most of the gaps are supposed to be repetitive patterns because repetitive contigs were masked before scaffolding. There is a module of SOAPdenovo called GapCloser which fills gaps in the assembled scaffolds. The main algorithm contains two steps:

1. ***Import and preprocess reads and scaffolds***. Scaffolds are sheared into contigs at gaps. All reads specified by the configuration file are imported into memory by two indexing tables for forward and reverse complementary reads respectively. The two tables are sorted in lexicographical order.
2. ***Contigs are being extended to fill gaps iteratively***. In a single round of extension, reads aligned to proper positions on contigs according to its insert size are called paired-end supporting reads, and are prioritized to be used. During the extension of each base, the allele indicated by over 80% of all supporting reads is selected. Or it would be defined as a difference and the current round of extension will be stopped.

In SOAPdenovo2’s GapCloser, besides enhancing the program’s ability to deal with longer sequencing reads data, we mainly changed the strategy for contig extension. Firstly, we tried to categorize the type of divergences. Some divergences are caused by sequencing errors and others might be related to reads from repetitive regions. If a read contains more than two positions with bases that are inconsistent with the bases already chosen in the extended region, the read will be removed. Thus, additional divergences caused by the same reads should be avoided. Secondly, if a difference still cannot be solved by removing repetitive reads, we tried to recover all related reads crossing the differing base, including reads not only found in this round of extension, but also reads found in previous rounds and reads found in following rounds, This means that differences that remained in previous rounds of extension will be revised recursively.

1. **Evaluating the GAGE dataset**

GAGE is a comprehensive evaluation of genome assemblers [8]. It uses four real sequencing datasets including *S. aureus*, *R. sphaeroides*, Human Chromosome 14, and *B. impatiens*. The sequencing reads of Human Chromosome 14 were downloaded from a whole genome sequencing project (sequenced from cell line GM12878). Because we assembled an entire whole human genome named as ‘YH genome’ for the study, which requires more intensive computational resources and produces more representative results, we excluded the Human Chromosome 14 dataset to avoid repetition. All other three species were assembled with SOAPdenovo, SOAPdenovo2 and ALLPATHS-LG respectively. We then used the published GAGE evaluation pipeline to evaluate all the species.

1. **Updating the YH genome assembly**

We have sequenced the first Asian genome, known as the YH Genome, using Illumina HiSeq 2000 sequencing [9]. The details of the production are shown in Table S3. We sequenced approximately 34-fold overlapping paired-end reads that also makes the dataset optimized for ALLPATHS-LG. We assembled the genome with SOAPdenovo, SOAPdenovo2, SOAPdenovo2 multi-*k*-mer, SOAPdenovo2 sparse and SOAPdenovo2 sparse with multi-*k*-mer respectively to test the performance of SOAPdeonvo2 and each module. All the assembly parameters used are shown in Supplementary Table 6. We then mapped the assembly results to the human reference (GRCh37 major build) with LAST [10] to calculate the genome coverage. As shown in Table 2 of the main paper, the N50 scaffold of SOAPdenovo2 outperformed ALLPATHS-LG with an increase of more than 4-fold compared with SOAPdenovo. But the N50 contig of ALLPATHS-LG is the longest. However, the N50 contig could be further improved for SOAPdenovo2 by using 3’-end connected reads and a larger *k*-mer size than ALLPATHS-LG.

When running ALLPATHS-LG with default parameters, we encountered out of memory (OOM) errors on our 400 GB memory machine at the FixLocal module. Since machines with larger memory are extremely expensive and we were not able to get access to machines with larger memory, we disabled the FixLocal module by parameter “FIX_LOCAL=False” when running ALLPATHS-LG.

1. **Evaluation of the YH genome**

To check the characteristics of the assembled YH genome by SOAPdenovo2 compared with SOAPdenovo, we aligned the assembled YH genome to the human reference genome (GRCh37 major build) with LAST and found that approximately 96% of the novel assembled regions are repetitive sequences. Because the genome coverage is not increased significantly when using SOAPdenovo with the new dataset, the novel assembled repetitive sequences should attribute to the algorithm improvement of SOAPdenovo2 rather than because of the dataset itself. Based on the alignment, we examined the low coverage and fragmented genes assembled by SOAPdenovo mentioned in a previous study [11]. The results are shown in Table S4. The coverage of most of the genes were increased and the fragmented genes now have drastically decreased numbers of fragments.

We also aligned the published human specific segmental duplications (SD) sequences [12] to the assembled sequences of SOAPdenovo2 with LAST and found that both the coverage and copy number of SD sequences were increased. However, as shown in Table S5, there were still up to 47% SD sequences being covered only once, which is largely limited by the sequencing data instead of the assembly algorithms.

1. **Machine used**

We used a single computing node with 2 hexa-core Intel Xeon E5-2620 @2.00GHz and 400 GB memory. The system cache was cleaned with command “sysctl –w vm.drop_caches=3” before every experiment.

### Supplementary Tables

**Table S1** **Error correction results of 30X, 0.5% error rate simulated reads from *Arabidopsis thaliana*.** All programs were run using default parameters. FP, FN and TP stand for ‘false positive’, ‘false negative’ and ‘true positive’ respectively. The metrics are: 1) Trimmed error rate - number of error-bases trimmed divided by total number of error bases, 2) FN – error bases not being corrected, 3) FP – correct bases being modified to incorrect base, 3) TP – error bases being corrected, 4) Sensitivity - $TP\div(TP+FN)$, 5) Gain – $(TP-FP)\div(TP+FN)$.

| **Program** | **Reads left** | **Remaining error rate** | **Trimmed error rate** | **FN** | **FP** | **TP** | **Sensitivity** | **Gain** |
| --- | --- | --- | --- | --- | --- | --- | --- | --- |
| SOAPec-v1.0 | 95.40% | 0.02% | 26.98% | 2.77% | 0.89% | 99.11% | 97.23% | 96.36% |
| SOAPec-v2.0 | 99.74% | 0.01% | 38.80% | 2.91% | 0.64% | 99.36% | 97.09% | 96.47% |
| SOAPec-v2.0-DuoKmer* | 99.77% | 0.01% | 38.45% | 2.129% | 0.20% | 99.80% | 97.87% | 97.68% |
| Quake-v0.3.4 | 99.55% | 0.01% | 16.79% | 2.28% | 0.42% | 99.58% | 97.72% | 97.31% |

* For duo-kmer mode in SOAPec v2.0, we used consecutive *k*-mer length 17bp and space *k*-mer length 17 bp.

**Table S2** **Computational resources consumption of error correction programs**

| **Programs** | **Frequency Table Construction** | | **Correction** | |
| --- | --- | --- | --- | --- |
|  | **Memory (GB)** | **Time (Min)** | **Memory (GB)** | **Time (Min)** |
| SOAPec-v1.0 | 16.78 | 34.27 | 40.25 | 103.35 |
| SOAPec-v2.0 | 16.82 | 4.75 | 2.76 | 8.03 |
| SOAPec-v2.0-DuoKmer | 16.78 | 10.32 | 4.86 | 13.86 |
| Jellyfish* & Quake-v0.3.4 | 8.73 | 4.29 | 2.46 | 98.7 |

* Jellyfish [13] is a program to calculate the k-mer frequency of sequencing reads, and we used this program to construct k-mer frequency table for Quake as recommended.

**Table S3** **Summary of the production of the new YH dataset.** Physical depth is calculated using the whole spanning area of the paired-end reads.

| **Insert size (bp)** | **Read length (bp)** | **Sequencing depth** | **Physical depth** |
| --- | --- | --- | --- |
| 178, 484 | 100 | 41.6 | 51.9 |
| 2k | 90 | 3.4 | 51.1 |
| 5k | 90 | 2.8 | 90.5 |
| 10k | 90 | 5.0 | 309.9 |
| 20k | 44, 90 | 3.6 | 481.7 |
| 40k | 44 | 0.2 | 87.9 |

**Table S4** **Coverage of published SD sequences of YH genome**

| **Version** | | **Total number of sequences** | **Total matched** | | **50% Coverage** | | **90% Coverage** | | |
| --- | --- | --- | --- | --- | --- | --- | --- | --- | --- |
|  |  |  | **Number** | **Percentage** | **Number** | **Percentage** | | **Number** | **Percentage** |
| v1 | 1 cover | 8,595 | 8,587 | 99.91 | 6,491 | 75.52 | | 1,851 | 21.54 |
|  | Multi cover |  | 8,587 | 99.91 | 368 | 4.28 | | 2 | 0.02 |
| v2 | 1 cover |  | 8,595 | 100 | 8,587 | 99.91 | | 8,514 | 99.06 |
|  | Multi cover |  | 8,595 | 100 | 6,641 | 77.27 | | 4,522 | 52.61 |

**Table S5** **Coverage and fragments on repetitive genes of YH genomes**. Comparing version 2 with version 1 assembly, the coverage of all the previously fragmented genes has increased. Nine out of 10 genes that were previously missing can now be partially covered.

| **Gene symbol** | **Length** | **Copy number** | **Type** | **YH version 1 [11]** | | **YH version 2** | |
| --- | --- | --- | --- | --- | --- | --- | --- |
|  |  |  |  |  |  |  |  |
|  |  |  |  | **Fragments** | **Coverage (%)** | **Scaffold number** | **Coverage (%)** |
| HYDIN | 423,280 | 3.47 | Fragmented | 215 | 95.82 | 5 | 97.42 |
| PRIM2 | 330,953 | 3.87 | Fragmented | 213 | 82.3 | 12 | 98.33 |
| CNTNAP3 | 215,534 | 4.65 | Fragmented | 208 | 84.92 | 61 | 63.6 |
| CDH12 | 1,102,757 | 1.87 | Fragmented | 184 | 95.86 | 4 | 99.93 |
| GRM5 | 561,389 | 2.11 | Fragmented | 162 | 90.4 | 4 | 96.81 |
| TYW1 | 242,679 | 3.27 | Fragmented | 155 | 82.94 | 2 | 100 |
| PARG | 345,007 | 4.17 | Fragmented | 154 | 57.14 | 3 | 99.94 |
| PDE4DIP | 124,318 | 7.4 | Fragmented | 147 | 93.31 | 12 | 4.11 |
| DPP6 | 936,219 | 1.93 | Fragmented | 146 | 80.46 | 22 | 97.82 |
| NOTCH2 | 158,098 | 2.97 | Fragmented | 142 | 95.26 | 3 | 54.64 |
| FAM90A7 | 18,864 | 36.03 | Missing | 0 | 0 | 2 | 32.61 |
| NPIP | 14,631 | 30.73 | Missing | 0 | 0 | 9 | 32.66 |
| LOC100132832 | 13,558 | 19.82 | Missing | 0 | 0 | 4 | 79.56 |
| FAM86B2 | 10,726 | 20.82 | Missing | 0 | 0 | 7 | 80.6 |
| LOC440295 | 9,401 | 27.21 | Missing | 0 | 0 | 3 | 20.04 |
| LOC442590 | 9,329 | 32.2 | Missing | 0 | 0 | 4 | 58.78 |
| WBSCR19 | 9,233 | 32.98 | Missing | 0 | 0 | 4 | 59.28 |
| DUX4 | 8,204 | 195.66 | Missing | 0 | 0 | 0 | 0 |
| GSTT1 | 8,145 | 0.44 | Missing | 0 | 0 | 0 | 0 |
| REXO1L1 | 7,031 | 134.92 | Missing | 0 | 0 | 2 | 42.27 |

* These genes are listed in paper [11]. We evaluated these genes in the old and updated version of the YH assembly.

**Table S6** **The parameters used in SOAPdenovo2 pipeline for the YH assembly**

| **Program** | **Modules** | **Commands** |
| --- | --- | --- |
| SOAPfilter | - | perl makeSH.pl -q 64 -f 0 -y -z -p -b lane.lst lib.lst && sh lane.lst.filter.sh |
| SOAPec | kmerfreq | KmerFreq_HA_v2.0 -k 23 -f 0 -t 24 -b 1 -i 400000000 -l read.lst -p YH_k23 |
|  | correction | Corrector_HA_v2.0 -k 23 -l 2 -e 1 -w 1 -q 30 -r 45 -t 24 -j 1 -Q 64 -o 1 YH_k23.freq.gz read.lst |
| SOAPdenovo | pregraph | SOAPdenovo-63mer_v2.0 pregraph -K 45 -s all_2.0.cfg -o asm_45 -p 24 |
|  | contig | SOAPdenovo-63mer_v2.0 contig -s all_2.0.cfg -g asm_45 -m 61 -M 2 -e 1 -p 24 |
|  | map | SOAPdenovo-63mer_v2.0 map -s long.cfg -g asm_45 -k 45 -p 24; (readslength >44bp)  SOAPdenovo-63mer_v2.0 map -s short.cfg -g asm_45 -k 31 -p 24; (readslength<44bp) |
|  | scaff | SOAPdenovo-63mer_v2.0 scaff -g asm_45 -p 24 -F |
|  | gapcloser | GapCloser_v1.12 -a asm_45.scafSeq -b gap_2.0.cfg -o asm_45.scafSeq.GC -p 31 -t 24 |

*All the programs mentioned here are included in the package of SOAPdenovo2.

**Supplementary Figures**

**Figure S1** **An illustration of co-op between Consecutive *k*-mer and Space *k*-mer**

**Figure S2** **An example of base correction by FAST approach**. Using *k*-mer (*k* in length), ideally a base error on a read will cause *k* continuous low frequency *k*-mers, these low frequency *k*-mers together are called a low frequency block in a read.

**Figure S3** **An illustration of base correction by DEEP approach**

**Figure S4** **The workflow of building sparse DBG in SOAPdenovo2**

DBG, *de Bruijn* graph

**Figure S5** **The contig type distribution of Human X Chromosome and *Arabidopsis thaliana*.** We simulated 60X of 100 bp paired-end reads and assembled to contigs with SOAPdenovo2 using a 31 bp *k*-mer size. Then we mapped the contigs to the reference genome and categorized the contigs into four types: ‘error contig’, ‘unique contig’, ‘similar contig’ and ‘repeat contig’. The x-axis shows the length of the mapped contigs. With the different gradient of ‘repeat contigs’ between Human X Chromosome and *Arabidopsis*, the contig length distribution also varies. Because of including more short repetitive patterns along the whole genome, *Arabidopsis* has a relatively shorter N50 contig than the Human X Chromosome.


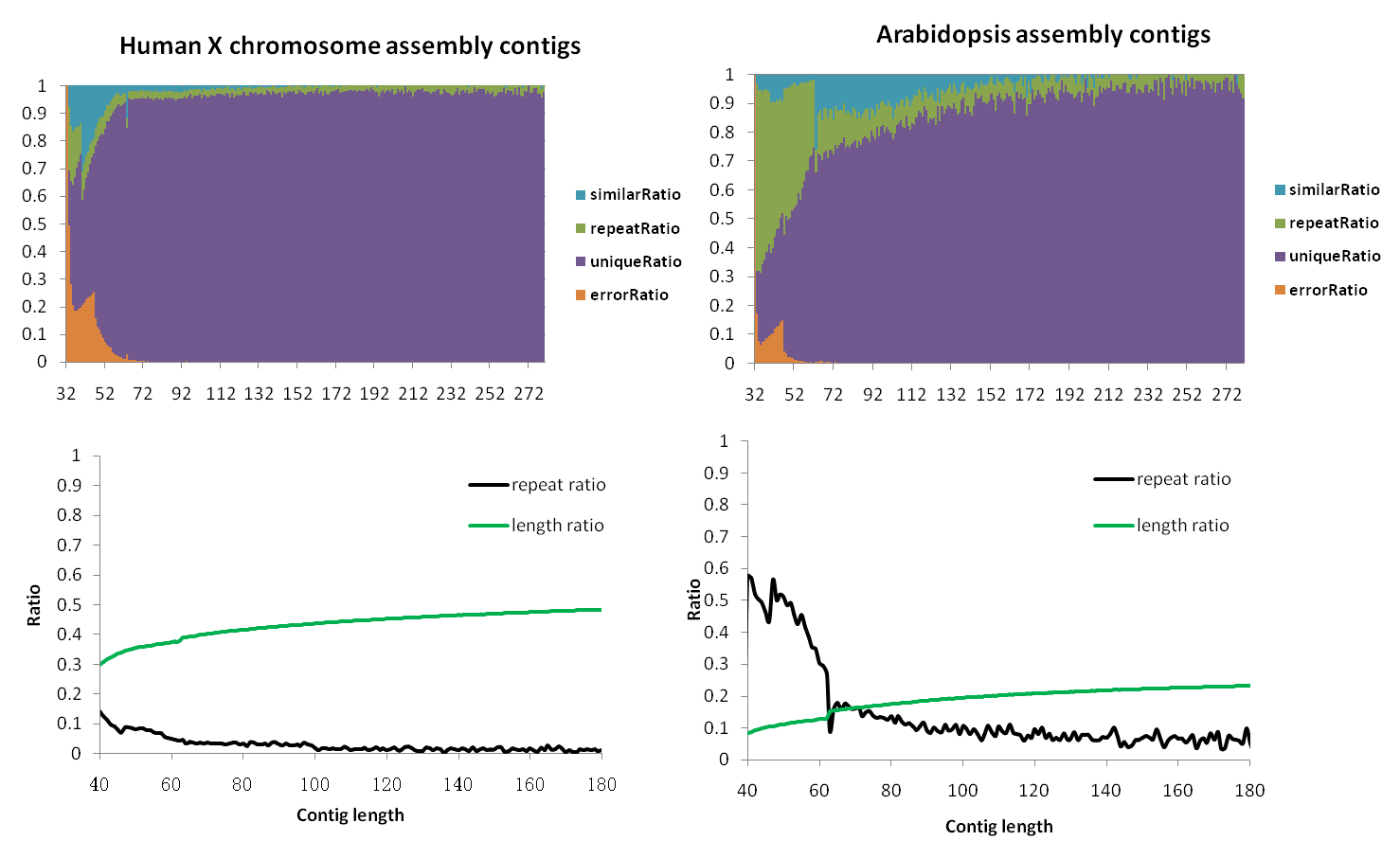


**Figure S6** **A theoretical topological structure of heterozygous contig pairs.** H1 and H2 contigs are a pair of heterozygous contigs. They have similar relationships to the other adjacent contigs as revealed by paired-end reads (Start contig and End contig). As a result, they would be located at approximately the same position in the scaffold, causing a divergence and stopping the scaffold form extension.


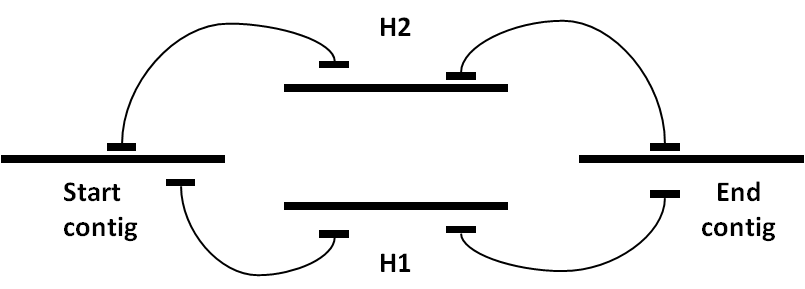


**Figure S7** **The detection and rectification of chimeric scaffolds** **(A)** Two sets of contigs contain a similar repetitive contig (red). **(B)** Chimeric scaffold due to the lack of link support between repeat contig, the blue contig on the left, and the green contig on the right. **(C)** The green contig on the left side of repeat contig has links to another scaffold (green), while the blue contig on the right side of repeat contig has links to another scaffold (blue) too. **(D)** Two revised scaffolds without repeat contig.

**References**

1. Hu X, Yuan J, Shi Y, Lu J, Liu B, Li Z, Chen Y, Mu D, Zhang H, Li N, Yue Z, Bai F, Li H, Fan W: **pIRS: Profile-based Illumina pair-end reads simulator.** *Bioinformatics* 2012, **28:**1533-1535.

2. Kelley D, Schatz M, Salzberg S: **Quake: quality-aware detection and correction of sequencing errors.** *Genome Biol* 2010, **11:**R116.

3. Ye C, Ma ZS, Cannon CH, Pop M, Yu DW: **Exploiting sparseness in de novo genome assembly.** *BMC Bioinformatics* 2012, **Suppl 6:**S1.

4. Li Z, Chen Y, Mu D, Yuan J, Shi Y, Zhang H, Gan J, Li N, Hu X, Liu B, Yang B, Fan W: **Comparison of the two major classes of assembly algorithms: overlap–layout–consensus and de-bruijn-graph.** *Brief Funct Genomics* 2012, **11:**25-37.

5. Liu B, Yuan J, Yiu S, Li Z, Xie Y, Chen Y, Shi Y, Zhang H, Li Y, Lam T, Luo R: **COPE: An accurate k-mer based pair-end reads connection tool to facilitate genome assembly.** *Bioinformatics* 2012, **28:**2870-2874.

6. Peng Y, Leung HC, Yiu SM, Chin FY: **IDBA-UD: A de Novo Assembler for Single-Cell and Metagenomic Sequencing Data with Highly Uneven Depth.** *Bioinformatics* 2012, **28:**1420-1428.

7. Li R, Zhu H, Ruan J, Qian W, Fang X, Shi Z, Li Y, Li S, Shan G, Kristiansen K, Li S, Yang H, Wang J, Wang J: **De novo assembly of human genomes with massively parallel short read sequencing.** *Genome Res* 2010, **20:**265-272.

8. Salzberg SL, Phillippy AM, Zimin A, Puiu D, Magoc T, Koren S, Treangen TJ, Schatz MC, Delcher AL, Roberts M, Marçais G, Pop M, Yorke JA: **GAGE: A critical evaluation of genome assemblies and assembly algorithms.** *Genome Res* 2012, **22:**557-567.

9. Wang J, Wang W, Li R, Li Y, Tian G, Goodman L, Fan W, Zhang J, Li J, Zhang J, Guo Y, Feng B, Li H, Lu Y, Fang X, Liang H, Du Z, Li D, Zhao Y, Hu Y, Yang Z, Zheng H, Hellmann I, Inouye M, Pool J, Yi X, Zhao J, Duan J, Zhou Y, Qin J *et al*: **The diploid genome sequence of an Asian individual.** *Nature* 2008, **456:**60-65.

10. Kiełbasa SM, Wan R, Sato K, Horton P, Frith MC: **Adaptive seeds tame genomic sequence comparison.** *Genome Res* 2011, **21:**487-493.

11. Alkan C, Sajjadian S, Eichler EE: **Limitations of next-generation genome sequence assembly.** *Nat Methods* 2011, **8:**61-65.

12. She X, Jiang Z, Clark RA, Liu G, Cheng Z, Tuzun E, Church DM, Sutton G, Halpern AL, Eichler EE: **Shotgun sequence assembly and recent segmental duplications within the human genome.** *Nature* 2004, **431:**927-930.

13. Marçais G, Kingsford C: **A fast, lock-free approach for efficient parallel counting of occurrences of k-mers.** *Bioinformatics* 2011, **27:**764-770.
